# Detection and identification of *Mucorales* and *Aspergillus* in paraffin-embedded samples by real-time quantitative PCR

**DOI:** 10.3389/fcimb.2023.1082347

**Published:** 2023-03-02

**Authors:** Xiaolin Jiang, Yong Jiang, Feng Ye

**Affiliations:** ^1^ Department of Pathology, West China Hospital of Sichuan University, Chengdu, China; ^2^ Department of Pathology, Guangyuan Central Hospital, Guangyuan, China

**Keywords:** *mucorales*, *aspergillus*, FFPE, RQ-PCR, 18SrRNA gene, 28SrRNA gene

## Abstract

**Background:**

In this study, we used real-time quantitative PCR (RQ-PCR) to rapidly detect *Mucorales* and *Aspergillus* in formalin-fixed, paraffin-embedded (FFPE) samples, targeting 18SrRNA gene and 28SrRNA gene. Identification of *Mucorales* and *Aspergillus* was analysed by combining *Mucorales* RQ-PCR (*Mucorales*18SrRNA and *Mucorales*28SrRNA) with *Aspergillus* RQ-PCR (*Aspergillus*18SrRNA and *Aspergillus*28SrRNA).

**Objectives:**

The aims of this study were to compare the diagnostic performances of four RQ-PCR assays as single and combined diagnostic and identification tools.

**Methods:**

We collected 12 control group samples and 81 experimental group samples diagnosed by histopathology, including mucormycosis (19 patients, 21 FFPE samples), aspergillosis (54 patients, 57 FFPE samples) and mucormycosis with aspergillosis (3 patients, 3 FFPE samples). All samples were detected by four RQ-PCR tests to compare and analyze diagnostic performance.

**Results:**

The sensitivities of *Mucorales*18SrRNA and *Mucorales*28SrRNA were both 75%, with the tests having specificities of 97.10% and 94.20%. The sensitivities of *Aspergillus*18SrRNA and *Aspergillus*28SrRNA were 73.33% and 65%, with the tests having specificities of 87.88% and 81.82%. The values of the evaluation indexes of the combined detection of *Mucorales*28SrRNA and *Aspergillus*18SrRNA (M28A18) were the highest with a kappa coefficient value of 0.353, followed by M18A18. M28A18 had a sensitivity of 67.90% and a specificity of 100%.

**Conclusions:**

We recommend using the combination of *Mucorales* RQ-PCR and *Aspergillus* RQ-PCR as a screening tool to detect samples suspected of mucormycosis and/or aspergillosis.

## Introduction

Over the past few decades, the incidence of invasive mold disease (IMD) has increased significantly and the fungal spectrum of IMD has broadened. According to literature, over 100,000 IMD cases occur each year, and these are associated with high morbidity and mortality in immunocompromised patients who have hematological malignancy and transplantation (Ruiz-Camps and Jarque, 2014; Ruhnke et al., 2015; Pegorie et al., 2017; Springer et al., 2018).

The members of the order *Mucorales* and genus *Aspergillus* are the most common opportunistic pathogens of IMD (Oren and Paul, 2014). Because of the significantly different antifungal susceptibilities and the complexity of the population of patients at risk, management of patients with IMD which lacks typical clinical manifestations has become increasingly complex. Therefore, early and reliable diagnostic methods are necessary for effective treatment.

Currently, the gold standard for the diagnosis of invasive fungal infections depends on histopathology and culture. However, culture is time-consuming and may fail if the potential microbial causes are not considered during sample collection, so formalin-fixed, paraffin-embedded (FFPE) bioptic material is collected for subsequent histological diagnosis. FFPE tissues obtained from patients with proven IMDs are frequently used to detect the etiology of invasive mycoses (Tarrand et al., 2003; Munoz-Cadavid et al., 2010; Babouee Flury et al., 2014). While histopathology can prove invasive fungal infections, the analytical correctness of histological findings is no more than 79% (Sangoi et al., 2009). Therefore, preliminary histological results should be interpreted cautiously (Guarner and Brandt, 2011) and supported by the culture whenever possible. In addition, histopathological observations of fungal shape and arrangement may not be sufficient for accurate identification of the *Mucorales* and *Aspergillus* if only a limited quantity of fungal hyphae is present.

In recent years, considerable efforts have been made to develop more sensitive and specific tools and protocols for IMD diagnosis. It is reported that PCR-based techniques, including conventional, semi-nested and real-time PCR, can be used to identify fungal agents in FFPE tissue (Bialek et al., 2005; Rickerts et al., 2006; Walsh et al., 2011; Springer et al., 2016a). RQ-PCR is very suitable for detecting the DNA of FFPE samples which are easily degraded. There are reports using the 18SrRNA gene and the 28SrRNA gene regions to detect and distinguish mucormycosis and invasive aspergillosis (Bialek et al., 2005; Walsh et al., 2011; Springer et al., 2016a; Gade et al., 2017).

The objective of this study was to evaluate RQ-PCR protocols by the use of TaqMan technology for detection and identification of *Mucorales* and *Aspergillus* in FFPE samples, targeting the 18SrRNA gene and the 28SrRNA gene. Identification of *Mucorales* and *Aspergillus* was analyzed by the combination of *Mucorales* RQ-PCR (*Mucorales*18SrRNA and *Mucorales*28SrRNA) and *Aspergillus* RQ-PCR (*Aspergillus*18SrRNA and *Aspergillus*28SrRNA).

## Materials and methods

### Samples

Ethical approval was obtained from the West China Hospital Ethics Committee of Sichuan University. According to local ethics, we have applied for exemption from written informed consent. We collected 81 experimental group samples (from 76 patients) in the Department of Pathology of West China Hospital from January 2015 to January 2018 with positive histopathology results, including mucormycosis (19 patients, 21 FFPE samples), aspergillosis (54 patients, 57 FFPE samples) and mucormycosis with aspergillosis (3 patients, 3 FFPE samples).

In addition, 12 FFPE tissue specimens from patients were used as controls including 6 without IMDs and 6 with other fungal infections. All of the slides including hematoxylin-eosin (H&E), periodic acid-Schiff (PAS) and/or Gomori-methenamine-silver (GMS) from each patient were reviewed and confirmed according to European Organization for Research on Treatment of Cancer and the Mycoses Study Group (EORTC/MSG) (De Pauw et al., 2008) by two professional and experienced pathologists with consistent diagnosis independently and in duplicate.

Isolates of laboratory strains included *Aspergillus flavus*, *Aspergillus fumigatus*, *Rhizomucor miehei*, *Candida albicans*, *Cryptococcus neoformans*, and *Fusarium oxysporum*. All isolated strains were provided by the Clinical Microbiology Laboratory, West China Hospital of Sichuan University.

### DNA extraction

Four FFPE tissue sections with 4-to-5-um from each specimen were used for DNA extraction. Each section was cut at a different position of the disposable knife of the microtome to prevent DNA cross-contamination due to attached cells at that position of the knife from one section to the next. If the sample surface was exposed to air, discard the first 2–3 sections. For deparaffinization, the sections were put into 1.5 mL tubes. 1 ml of xylene was added, centrifuged at full speed for 2 minutes at room temperature and discarded the supernatant by pipetting. Then, 1 ml ethanol was added, centrifuged and discarded like xylene. After incubation of the tissue at 37°C to evaporate the remains of the ethanol, DNA extraction was performed according to the manufacturer’s instructions for the QIAamp DNA FFPE tissue kit (Qiagen, Germany) with the following modifications. All FFPE tissue samples were incubated over night in proteinase K and ATL buffer at 56°C. Fungal cell walls were lysed by *Arthrobacter luteus* lyticase L2524 (Sigma, USA) for 45minutes at 37°C. The DNA was eluted with 100 µl Buffer ATE and stored at -20°C.

DNA extraction of laboratory strains was performed according to the manufacturer’s instructions for the QIAamp DNA Mini kit (Qiagen, Germany) with the following modifications. Fungal cell walls were lysed by *Arthrobacter luteus* lyticase L2524 (Sigma, USA) for 30 minutes at 30°C, incubated 1 to 3 hours in proteinase K and ATL buffer at 56°C. The DNA was stored at -20°C.

### Real-time PCR assays

**Figure 1:**
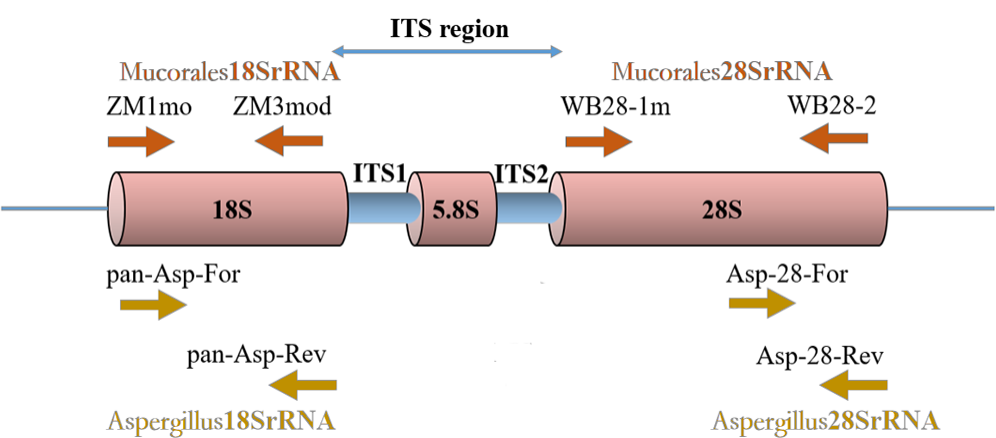
Locations of four real-time PCR assays target. An rDNA single repeat is shown. The primers ZM1mo and ZM3mod of *Mucorales*18SrRNA assay target 18SrRNA gene region. The primers WB28-1m and WB28-2 of *Mucorales*28SrRNA assay target 28SrRNA gene region. The primers pan-Asp-For and pan-Asp-Rev of *Aspergillus*18SrRNA assay target 18SrRNA gene region. The primers Asp-28-For and Asp-28-Rev of *Aspergillus*28SrRNA assay target 28SrRNA gene region.

*Mucorales* RQ-PCR primers and probes targeting the 18SrRNA gene and the 28SrRNA gene were described by Springer et al. (Springer et al. 2016a). *Aspergillus* RQ-PCR primers and probes targeting the 18SrRNA gene were described by Walsh et al. (Walsh et al., 2011). The protocols of RQ-PCR amplifications were performed as described previously (Walsh et al., 2011; Springer et al. 2016a) with the exception of the design of a new primer pair. For optimal design of new primers and probes, multiple sequence alignments of *Aspergillus* 28SrRNA gene (National Center for Biotechnology Information [NCBI] public genetic database [GenBank]) were created using Geneious software (Biomatters, Auckland, New Zealand). Primer Express 3.0 (Applied Biosystems, Foster City, CA) was used to help select primers and probes of optimal melting temperatures (**Table 1**). The primers and probes of *Aspergillus* 28SrRNA were verified for its specificity by six laboratory isolated strains and normal human DNA, which thermocycling protocol are the same as *Aspergillus* 18SrRNA. The locations of real-time PCR assay targets are shown in **Figure 1**.

**Table 1:** Primers and probes.

Assay	Primer or probe	Primer sequence (5’-3’)
***Mucorales*18SrRNA**	Forward primer	TTACCRTGAGCAAATCAGARTG
	Reverse primer	AATCYAAGAATTTCACCTCTAGCG
	Probe	TYRR(G)G(G)B(A)T(T)T(G)T(A)TTT
***Mucorales*28SrRNA**	Forward primer	TTTGGGAATGCAGCCT
	Reverse primer	TCARAGTTCTTTTCAWCTTTCCCT
	Probe	CGARARACCGATAGCRAACAAGTACCGT
***Aspergillus*18SrRNA**	Forward primer	GTGGAGTGATTTGTCTGCTTAATTG
	Reverse primer	TCTAAGGGCATCACAGACCTGTT
	Probe	CGGCCCTTAAATAGCCCGGTCCG
***Aspergillus*28SrRNA**	Forward primer	CACTAGCCGGGCAACCG
	Reverse primer	GACAGTCAGATTCCCCTTGTC C
	Probe	GCGGGCGCTTAACGACCAACTTAG

Parentheses indicate nucleotide with locked nucleic acid modification.

Nucleotides in upper case are wobble nucleotides: R stands for a or g; W for a or t; Y for c or t; B for g, c or t.

Both probes are FAM-labelled at the 5’ end and BHQ1 at the 3’ end.

We test all samples in triplicate. Amplification had to be reproducible, occurring in all 3 replicate wells, for a sample to be considered RQ-PCR positive. The positivity cutoff of *Mucorales*18SrRNA and *Mucorales*28SrRNA was defined as both wells having Cq values of <36. The positivity cutoff of *Aspergillus*18SrRNA and *Aspergillus*28SrRNA was defined as both wells having Cq values of <35 and <33, respectively. To validate the presence of amplifiable DNA and absence of inhibitory substances, a PCR in FFPE samples was performed using the primer set G1 and G2 targeting the human β globin gene (Bialek et al., 2005). When the result was negative, DNA extraction was repeated if enough material was available. All primers and probes were synthesized in Sangon Biotech (Sangon, Shanghai, China).

For all assays, RQ-PCR amplifications were performed in a 25 ul mixture using a StepOnePlus thermocycler (Applied Biosystems). Each reaction mixture contained 12.5 ul TaqMan Universal PCR Master Mix (Applied Biosystems), 900 nM forward and reverse primer, 200 nM probe and 5 ul extracted DNA. The DNA extracted from *Rhizomucor miehei* and *Aspergillus flavus* were serially diluted and tested for each RQ-PCR assay to determine the limit of detection (LoD). In each run, negative (FFPE tissue specimen without IMD) and positive (isolated strains of *Rhizomucor miehei and Aspergillus flavus*) controls were included.

Identification of *Mucorales* and *Aspergillus* was analyzed by *Mucorales* RQ-PCR in combination with *Aspergillus* RQ-PCR, including *Mucorales*18SrRNA and *Aspergillus*18SrRNA (M18A18), *Mucorales*18SrRNA and *Aspergillus*28SrRNA (M18A28), *Mucorales*28SrRNA and *Aspergillus*18SrRNA (M28A18), and *Mucorales*28SrRNA and *Aspergillus*28SrRNA (M28A28). True positives of *Mucorales* RQ-PCR in combination with *Aspergillus* RQ-PCR were defined as cases proven according to the following criteria: for mucormycosis samples, *Mucorales* RQ-PCR positivity and *Aspergillus* RQ-PCR negativity; for mucormycosis with aspergillosis samples, both positivity; for aspergillosis samples, *Aspergillus* RQ-PCR positivity and *Mucorales* RQ-PCR negativity. Other results were regarded as negatives.

### Statistical analysis

All samples, including 81 experimental group samples and 12 control group samples, were detected by four RQ-PCR tests to compare and analyze the diagnostic performance, including negative predictive value (NPV), positive predictive value (PPV), sensitivity and specificity with likelihood ratios (LRs), and 95% confidence intervals (CIs) were calculated for NPV, PPV, sensitivity, and specificity. Cohen’s kappa coefficient was calculated to measure the agreement between any two assays. Statistical analysis was performed using SPSS, version 20 (SPSS, Chicago, IL, USA).

## Results

**Figure 2:**
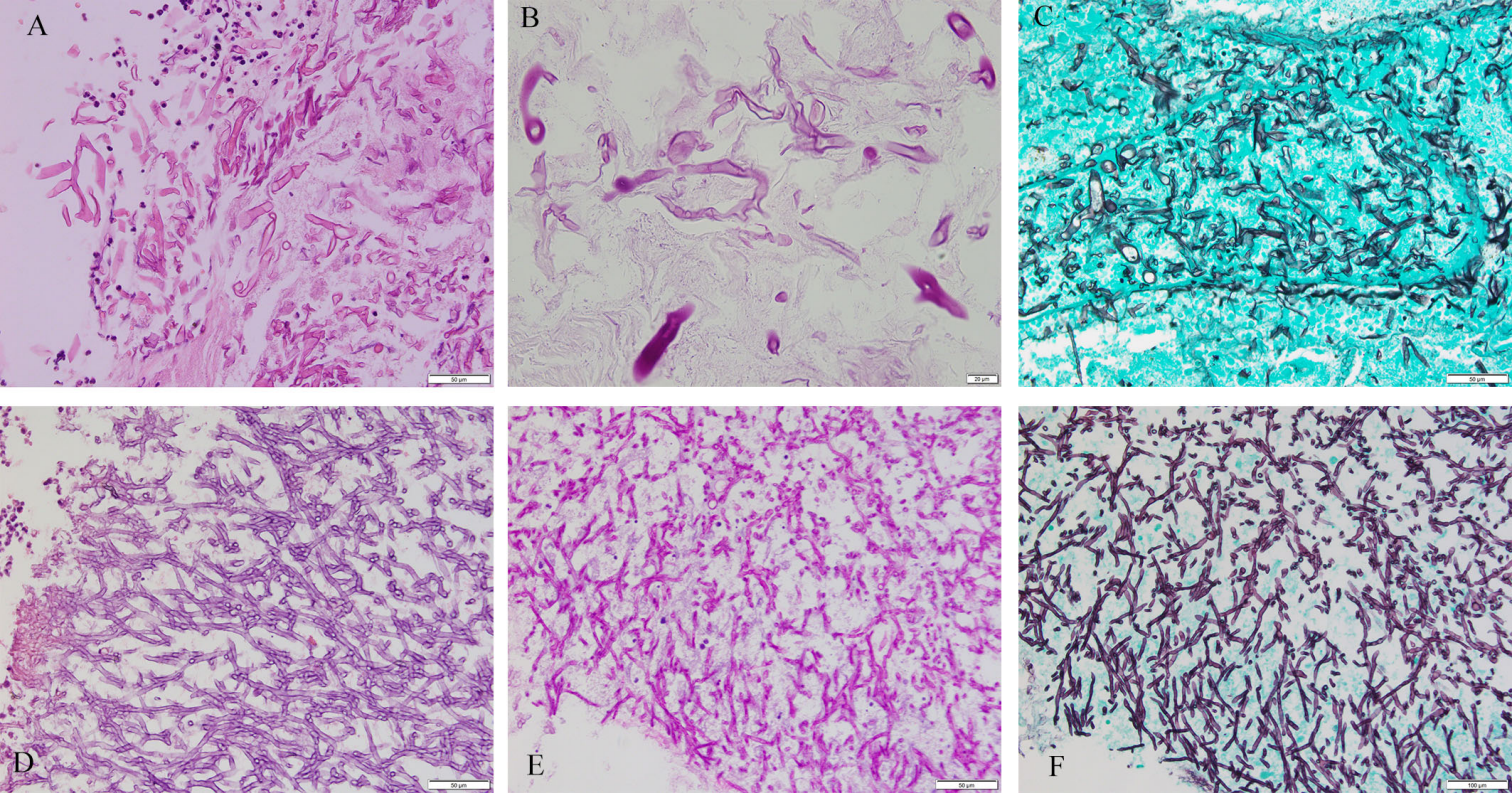
*Mucorales* and *Aspergillus*. Cytomorphology of *Mucorales* and *Aspergillus* in FFPE samples processed using the HE staining, PAS staining and GMS staining. The mycelium appeared magenta after PAS staining and brown-black after GMS staining. Magnification, 400×. **(A)** *Mucorales* with HE; **(B)** *Mucorales* with PAS; **(C)** *Mucorales* by GMS; **(D)** *Aspergillus* with HE; **(E)** *Aspergillus* with PAS; **(F)** *Aspergillus* with GMS. Lack of images of tissues with both *Aspergillus* and *Mucorales* infection.

The study involved 76 patients (F/M, 42/34; age, 50.95 ± 17.31 years) with following comorbidities: 23 (30.26%) had diabetes, 16 (21.05%) had hypertension, 10 (13.16%) had bronchiectasis, 8 (10.53%) had solid tumor, 7 (9.21%) had Chronic obstructive pulmonary disease (COPD), 5 (6.58%) had hematological malignancy, 5 (6.58%) had tuberculosis, and 16 (21.05%) had others. Slides stained with PAS or GMS were considered, respectively, positive if magenta or brown-black fungal hyphae with morphological features were observed (**Figure 2**). Positive special staining with GMS and/or PAS were 24/29 (82.76%). Positive culture cases were 12/52 (23.08%). Positive 1, 3-beta-D-glucan assay cases were 9/40 (22.50%). Positive Galactomannan assay cases were 12/42 (28.57%). The principal site of infections was in lungs (61 cases), next were in other sites (including one ileum, three nasal cavity, four maxillary sinus, two trachea, one external auditory canal, one toe and three main bronchus). CT of the chest was obtained in 61 patients with pulmonary infection. There was a wide spectrum of radiological findings, with the most common being 26 nodules, followed by 20 mass, 16 cavitation, 7 consolidation, 5 pleural effusion, and 3 air crescent sign. Result of bronchofibroscopy were obtained in 56/61 patients with pulmonary infection: 18 patients were normal, and other patients were mainly necrosis, luminal stenosis, and purulent secretion.

### Individual test performance

The LOD of *Mucorales*18SrRNA and *Mucorales*28SrRNA was 10^-1^copies/ul in *Rhizomucor miehei* DNA. In *Aspergillus*RQ-PCR assays, the LOD between *Aspergillus* 18SrRNA and *Aspergillus*28SrRNA was different (10^0^copies/ul vs. 10^1^copies/ul) for *Aspergillus flavus* DNA. The analytical specificity of *Aspergillus*28SrRNA assays was tested by adding genomic DNA from the six isolated strains. No cross-reactivity with non-*Mucorales* species or human DNA was observed. The specificity of *Mucorales*18SrRNA, *Mucorales*28SrRNA and *Aspergillus*18SrRNA have been tested in the previous articles (Walsh et al., 2011; Springer et al., 2016a).

**Figure 3:**
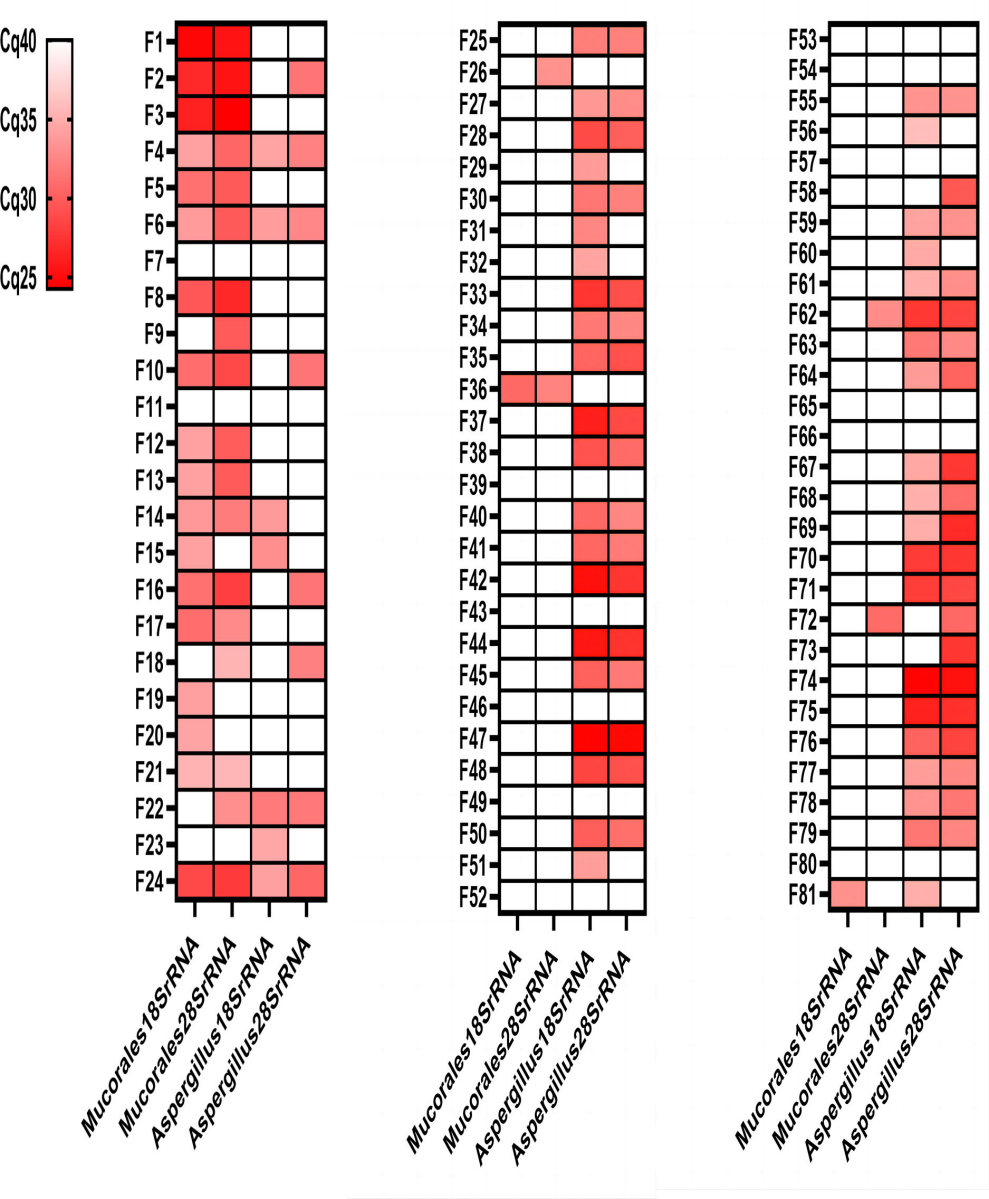
Genetic test results of the four RQ-PCR assays for 81 experimental group samples. Mucormycosis samples (F1-F21); Mucormycosis with Aspergillosis samples (F22-F24); Aspergillosis samples (F25-F81). Red: positive; White: negative.

Ninety-three different samples from 88 patients were analysed by the four different real-time PCR assays (**Figure 3**; **Table 2**). All control group samples were negative using the four different RQ-PCR assays. Thirteen experimental group samples were negative in all RQ-PCR assays.

**Table 2:** Four RQ-PCR testing results.

Samples (n)	*Mucorales*18SrRNA (%)	*Mucorales*28SrRNA(%)	*Aspergillus*18SrRNA (%)	*Aspergillus*28SrRNA (%)
**mucormycosis samples (n=21)**	17/21(80.95)	16/21(76.19)	4/21(19.05)	6/21(28.57)
**mucormycosis with aspergillosis samples (n=3)**	1/3(33.33)	2/3(66.67)	3/3(100)	2/3(66.67)
**aspergillosis samples (n=57)**	2/57(3.51)	4/57(7.02)	41/57(71.93)	37/57(64.91)
**control group samples (n=12)**	0/12	0/12	0/12	0/12

The *Mucorales*18SrRNA assay was positive in 17 of 21 mucormycosis samples (80.95%), 1 of 3 mucormycosis with aspergillosis samples (33.33%), and 2 of 57 aspergillosis samples (3.51%). The *Mucorales*28SrRNA assay behaved similarly, detecting 16 out of 21 mucormycosis samples (76.19%), 2 out of 3 mucormycosis with aspergillosis samples (66.67%), and 4 out of 57 aspergillosis samples (7.02%). Only two samples were negative by two different *Mucorales* RQ-PCR despite positive histology showed fungal hyphae (F7 and F11). The *Aspergillus*18SrRNA assay was positive in 41 of 57 aspergillosis samples (71.93%), 3 of 3 mucormycosis with aspergillosis samples (100%), and 4 of 21 mucormycosis samples (19.05%). The *Aspergillus*28SrRNA assay behaved similarly, detecting 37 out of 57 aspergillosis samples (64.91%), 2 out of 3 mucormycosis with aspergillosis samples (66.67%), and 6 out of 21 mucormycosis samples (28.57%). 12 control samples were negative in each assay. A concomitant infection was diagnosed in three samples by histopathology but verified by *Mucorales* RQ-PCR and *Aspergillus* RQ-PCR in two (F22 and F24). There were some “abnormal results” in our study, e.g., 2 of aspergillosis samples were positive by *Mucorales*18SrRNA (F36 and F81); 4 of the aspergillosis samples were positive by *Mucorales*28SrRNA (F26, F36, F62 and F72); 4 of mucormycosis samples were positive by *Aspergillus*18SrRNA (F4, F6, F14 and F15); 6 of mucormycosis samples were positive by *Aspergillus*28SrRNA (F2, F4, F6, F10, F16 and F18).

The sensitivity, specificity, PPV, NPV, positive and negative LRs of all four RQ-PCR assays are shown in **Table 3**. The sensitivities of *Mucorales*18SrRNA and *Mucorales* 28SrRNA were both 75%, with specificity, PPV, NPV, positive LR, and negative LR of *Mucorales*18SrRNA being 97.10%, 90.00%, 91.78%, 25.86, and 0.26, respectively. *Mucorales* 28SrRNA assay showed specificity of 94.20%, PPV of 81.82%, NPV of 91.55%, positive LR of 12.93, and negative LR of 0.27. The sensitivity, specificity, PPV and NPV of *Aspergillus*28SrRNA assays were lower than *Aspergillus*18SrRNA assays, for *Aspergillus*18SrRNA assays: 73.33%, 87.88%, 91.67%, and 64.54%, respectively; for *Aspergillus*28SrRNA assays: 65%, 81.82%, 86.67%, and 56.25%, respectively. The positive LR and negative LR of *Aspergillus*18SrRNA assays and *Aspergillus*28SrRNA assays were 6.05 and 0.30 vs. 3.58 and 0.43.

**Table 3:** Diagnostic performance of four RQ-PCR.

Assays	Sensitivity (%)	Specificity (%)	PPV (%)	NPV (%)	Positive LR	Negative LR
***Mucorales*18SrRNA**	75.00 (52.95, 89.40)	97.10 (88.99, 99.47)	90.00 (66.87, 98.24)	91.78 (82.35, 96.61)	25.86	0.26
***Mucorales*28SrRNA**	75.00 (52.95, 89.40)	94.20 (85.07, 98.13)	81.82 (58.99, 94.01)	91.55 (81.89, 96.52)	12.93	0.27
***Aspergillus*18SrRNA**	73.33 (60.11, 83.55)	87.88 (70.86, 96.04)	91.67 (79.13, 97.30)	64.45 (48.73, 77.71)	6.05	0.30
***Aspergillus*28SrRNA**	65.00 (51.52, 76.55)	81.82 (63.92, 92.38)	86.67 (72.51, 94.46)	56.25 (41.28, 70.23)	3.58	0.43

The sensitivities, specificities, positive predictive values, negative predictive values, likelihood ratios, and diagnostic odds ratios are displayed, with 95% confidence intervals being given in parentheses.

### Combined test performance

The true positive results of M18A18, M18A28, M28A18, and M28A28 were as follows: for mucormycosis samples, 13, 12, 13, and 10, respectively; for mucormycosis with aspergillosis samples, 1, 1, 2, and 2, respectively; for aspergillosis samples, 40, 37, 40, and 35, respectively. All combined tests were negative in control group samples (**Table 4**).

**Table 4:** The true positive results of *Mucorales* RQ-PCR in combination with *Aspergillus* RQ-PCR.

	mucormycosis samples (n=21)	mucormycosis with aspergillosis samples (n=3)	aspergillosis samples (n=57)	control group samples (n=12)	Total
**M18A18**	13	1	40	0	54
**M18A28**	12	1	37	0	50
**M28A18**	13	2	40	0	55
**M28A28**	10	2	35	0	47

A pairwise comparison of the tests showed that the highest level of agreement was M28A18, with a kappa coefficient value of 0.353. All other pairs of biomarkers showed less agreement (**Table 5**). Any combinations of *Mucorales* RQ-PCR assays and *Aspergillus* RQ-PCR assays had the same specificity (100%), PPV (100%), and positive LR (Infinity). The sensitivity, NPV, and negative LR were as follows: for M28A18, 67.90%, 31.58%, and 0.32 respectively; for M18A18, 66.67%, 30.77%, and 0.33, respectively; for M18A28, 61.73%, 27.91%, and 0.38, respectively; for M28A28, 58.02%, 26.09%, and 0.42, respectively. In all the samples, the values of the evaluation indexes of the combined detection of M28A18 were the highest, followed by M18A18.

**Table 5:** Diagnostic performance of dual *Mucorales* RQ-PCR and *Aspergillus* RQ-PCR testing.

	Sensitivity (%)	Specificity (%)	PPV (%)	NPV (%)	Positive LR	Negative LR	Kappa
**M18A18**	66.67 (55.22, 76.52)	100 (69.87, 100)	100 (91.73, 100)	30.77 (17.55, 47.73)	Infinity	0.33	0.340
**M18A28**	61.73 (50.22, 72.11)	100 (69.87, 100)	100 (91.11, 100)	27.91 (15.38, 43.90)	Infinity	0.38	0.294
**M28A18**	67.90 (56.49, 77.60)	100 (69.87, 100)	100 (91.87, 100)	31.58 (18.04, 48.79)	Infinity	0.32	0.353
**M28A28**	58.02 (46.54, 68.74)	100 (69.87, 100)	100 (90.59, 100)	26.09 (14.75, 41.41)	Infinity	0.42	0.263

## Discussion

The detection and identification of *Mucorales* and *Aspergillus* from FFPE samples played an important role in the diagnosis and management of aspergillosis and mucormycosis, whereas microscopy, serology, and culture were restricted by several disadvantages (Jensen et al., 1997; Frater et al., 2001; Rickerts et al., 2007; Hofman et al., 2010; Hamilos et al., 2011). Numerous RQ-PCR assays have been described for detection of *Mucorales* (Bernal-Martinez et al., 2013; Millon et al., 2013; Lengerova et al., 2014) and *Aspergillus* (Lass-Florl et al., 2011; Fricke et al., 2012; Paholcsek et al., 2014; Pini et al., 2015). In this study, we evaluated two *Mucorales* RQ-PCR assays, two *Aspergillus* RQ-PCR assays, and four combined tests to rapidly detect and identify *Mucorales* and *Aspergillus*.

More recently, the RQ-PCR of *Mucorales* from 268 serum samples and 12 FFPE samples was a promising test method with sensitivity of 91% targeting 18SrRNA gene (Springer et al., 2016b) and 86% targeting 28SrRNA gene (Springer et al., 2016a). In this study, two *Mucorales* RQ-PCR only had a sensitivity of 75%. The lower sensitivity may be relevant to small sample size and different sample types. The RQ-PCR detecting *Mucorales* also had a specificity of 100% in FFPE samples but a lower sensitivity of 56% by Hata et al. (Hata et al., 2008). The specificity of our two *Mucorales* RQ-PCR assays was 97.1% and 94.20%, respectively. As reported in the literature, the RQ-PCR specificity was 87.5% (Springer et al., 2016a). In *Mucorales* RQ-PCR assays, the diagnostic parameters and LoD values for the different assays indicated that *Mucorales*18SrRNA would provide the best diagnostic accuracy. These results support the findings of Springer et al. (Springer et al., 2016a; Springer et al., 2016b).

In our study, the specificity and sensitivity of *Aspergillus*18S rRNA were 87.88% and 73.33%, respectively, which is consistent with the results of previously published studies (Hadrich et al., 2011; Luong et al., 2011). The MycAssay™ *Aspergillus* real-time PCR kit was tested on tissues by the manufacturer with 15 different *Aspergillus* spp., including multiple strains of *Aspergillus fumigatus*, *Aspergillus niger*, *Aspergillus terreus*, and *Aspergillus nidulans*, having a sensitivity of 82% and a specificity of 79% (Lass-Florl et al., 2011). In our study, the specificity of two *Aspergillus* RQ-PCR assays was elevated, whereas sensitivity was reduced.

However, some limitations and several considerations indicate that it has some drawbacks in the *Mucorales* RQ-PCR or *Aspergillus* RQ-PCR alone. Our studies have evaluated the utility of detection of *Mucorales* RQ-PCR and *Aspergillus* RQ-PCR. Our finding, combining the results of these two tests gave optimal specificity and PPV, could be used to detect and identify *Mucorales* and *Aspergillus* and may provide a solution when gold standard tests were conflicting. Given the ubiquity of *Aspergillus* and *Mucorales* in the environment, combining *Mucorales* RQ-PCR with *Aspergillus* RQ-PCR would give clinicians greater confidence in detecting and identifying them at the same time and reduce the false positive and/or negative rate. The best combination was the M28A18, with a sensitivity of 67.90%, a specificity of 100%, which had the highest diagnostic potential with FFPE samples. This is inconsistent with the conclusion that *Mucorales*18SrRNA is better than *Mucorales*28SrRNA in diagnostic significance, which may be related to the small sample size. In all samples, the number of true positives of M18A18 was very similar to M28A18 (54 VS. 55). Their diagnostic significance needs to be further evaluated in future studies with larger sample sizes.

This study has several limitations, which may be the cause of some “abnormal results”. First, the sample size is too small, especially that the mucormycosis sample is only 21 cases from 19 patients. Due to the limited number of FFPE samples and strains, the very low number of *Aspergillus* and *Mucorales* species was tested, the number of *Aspergillus* and *Mucorales* species that can be detected by these primers needs to be further evaluated. Second, RQ-PCR assays require strictly positive and negative controls. The limitations of false positive and false negative errors due to amplification and contamination for assessing the value of a molecular diagnostic test have been eloquently highlighted (Mies, 1994; Cataloluk et al., 2003). In some samples, fluorescence signals higher than the positive Cq cutoff was detected, which may be caused by false negatives due to the low number of fungal hyphae. Third, the use of mold-active drugs may affect detection result. There are conflicting reports about the effect of antifungal therapy on the performance of tests. Antifungal therapy has been reported to both decrease (Reinwald et al., 2012) and increase (Musher et al., 2004) the diagnostic performance of RQ-PCR. Furthermore, “abnormal results” may be caused by a condition other than Mucormycosis and Aspergillosis or by drug treatment. Fourthly, Formalin fixed and paraffin wax embedded tissues can cause DNA degradation, only short sequences can be amplified from this type of tissue (Bonin et al., 2003). Although the amplified sequences of the four RQ-PCRs in this study were less than 200bp, which weakened the influence of DNA fragmentation, it may still reduce the sensitivity. Fifth, due to funding reasons, we did not use commercialized kits for the detection of *Mucorales* and *Aspergillus* DNA in clinical samples. Finally, Misclassification using these inconsistent criteria of RQ-PCR can occur for many reasons, e.g., detection of pathogenic fungi may be missed due to the diversity of fungal species and test samples, and there are multiple laboratorial protocols. Each suffers from different disadvantages such as vulnerability to contamination or limited detection of selected species or genera.

In conclusion, this preliminary study showed that the two *Aspergillus* RQ-PCR assays and two *Mucorales* RQ-PCR assays had high potential for the diagnosis of *Mucorales* and *Aspergillus* in FFPE samples. We envisage *Aspergillus* RQ-PCR and *Mucorales* RQ-PCR combination approach as a nearpatient test, allowing an immediate detection and identification of *Mucorales* and *Aspergillus*, with RQ-PCR results being available within a short time for samples of mucormycosis with aspergillosis. This combination approach can provide useful information when a small number of fungi are present, or the histological diagnosis is difficult in mucormycosis with aspergillosis samples. In the future, these assays may be used as a screening tool to detect other types of samples suspected of having mucormycosis and/or aspergillosis, such as serum, bronchoalveolar lavage fluid (BALF), and cytological samples. The results of our study should be validated in multicenter studies to develop tests for this clinical application.

## Data availability statement

The raw data supporting the conclusions of this article will be made available by the authors, without undue reservation.

## Ethics statement

The studies involving human participants were reviewed and approved by West China Hospital Ethics Committee of Sichuan University. Written informed consent for participation was not required for this study in accordance with the national legislation and the institutional requirements.

## Author contributions

YJ and FY and conceived and designed the experiments. XJ analyzed the data and wrote the manuscript. All authors contributed to the article and approved the submitted version.
